# Application of combined omics platforms to accelerate biomedical discovery in diabesity

**DOI:** 10.1111/nyas.12116

**Published:** 2013-05-09

**Authors:** Irwin J Kurland, Domenico Accili, Charles Burant, Steven M Fischer, Barbara B Kahn, Christopher B Newgard, Suma Ramagiri, Gabriele V Ronnett, John A Ryals, Mark Sanders, Joe Shambaugh, John Shockcor, Steven S Gross

**Affiliations:** 1 Department of Medicine, Stable Isotope and Metabolomics Core Facility, Albert Einstein College of Medicine Diabetes CenterBronx, New York; 2 Diabetes and Endocrinology Research Center, Columbia UniversityNew York, New York; 3 Department of Internal Medicine, University of Michigan Medical SchoolAnn Arbor, Michigan; 4 Agilent TechnologiesSanta Clara, California; 5 Beth Israel Deaconess Medical Center and Harvard Medical SchoolBoston, Massachusetts; 6 Daegu Gyeongbuk Institute of Science and TechnologyDaegu, Korea; 7 Sarah W. Stedman Nutrition and Metabolism Center, Duke University Medical CenterChapel Hill, North Carolina; 8 AB SCIEX, ConcordOntario, Canada; 9 Department of Neuroscience, Johns Hopkins University School of MedicineBaltimore, Maryland; 10 Metabolon, IncDurham, North Carolina; 11 Thermo Fisher ScientificSomerset, New Jersey; 12 Genedata IncSan Francisco, California; 13 Waters CorporationMilford, Massachusetts; 14 Department of Pharmacology, Weill Cornell Medical CollegeNew York, New York

**Keywords:** omics, diabesity, diabetes, obesity, metabolomics, proteomics, lipidomics, metabolism, metabolic profiling

## Abstract

Diabesity has become a popular term to describe the specific form of diabetes that develops late in life and is associated with obesity. While there is a correlation between diabetes and obesity, the association is not universally predictive. Defining the metabolic characteristics of obesity that lead to diabetes, and how obese individuals who develop diabetes different from those who do not, are important goals. The use of large-scale omics analyses (e.g., metabolomic, proteomic, transcriptomic, and lipidomic) of diabetes and obesity may help to identify new targets to treat these conditions. This report discusses how various types of omics data can be integrated to shed light on the changes in metabolism that occur in obesity and diabetes.

## Introduction

Diabetes is an increasing concern not only for Western countries, where diet and lifestyle promote expanding waistlines and insulin resistance, but also for developing countries in which the effects of changing diet on the health of their populations are already visible. In the U.S., diabetes affects approximately 11% of the population over age 20, and there are an additional 79 million adults with pre-diabetes, a condition that often precedes diabetes in which glucose levels are higher than normal.^1^

Diabetics suffer an impairment of the body's ability to switch between glucose and fat as energy sources. Normally, when a person has not eaten recently (a fasting state), the muscles preferentially oxidize fat over glucose to ensure a supply of glucose for the brain. After a person eats, however, there is excess glucose in the system, and the muscles switch their primary energy source and begin oxidizing glucose and storing fats. Even early in the evolution of diabetes (i.e., in the pre-diabetic state referred to as metabolic syndrome), individuals are unable to make this fuel switch, a physiological maladaptation termed *metabolic inflexibility*.^2^,^3^ Muscles that use too much glucose in the fasted state contribute to fasting hyperlipidemia, and muscles that continue to oxidize fats in the fed state, instead of switching to glucose utilization, contribute to post-prandial hyperglycemia. Muscle metabolic inflexibility, along with the failure of insulin to suppress fat breakdown and post-prandial hepatic glucose production in the pre-diabetic and diabetic states (insulin resistance), results in high blood lipid and glucose levels.

As diabetes and its associated comorbidities—such as cardiovascular disease, kidney disease, and neurological disorders—rise in epidemic proportions, it is now more important than ever to develop new tools to understand the complex metabolic mechanisms and pathways involved in this disease and to find new therapeutic targets. In April 2012, leaders in this field met at the New York Academy of Sciences to discuss how various types of omics data (metabolomic, proteomic, transcriptomic, and lipidomic) can be integrated to reveal a more complete picture of these mechanisms.

The primary focus of the conference “Application of Combined ‘omics Platforms to Accelerate Biomedical Discovery in Diabesity” was obesity-induced diabetes—*diabesity*, which covers a constellation of signs, including obesity, insulin resistance, metabolic syndrome, and diabetes.^4^,^5^ Not all obese people have diabetes and not all people with diabetes are obese, but there is definitely a connection between the two conditions. One of the main questions throughout the conference was how to use omics data to create a phenotypic profile of disease state progression in order to understand why some individuals develop diabetes and its associated complications, while others do not.

## New tools and frameworks for gathering and visualizing omics data

**Figure 1:**
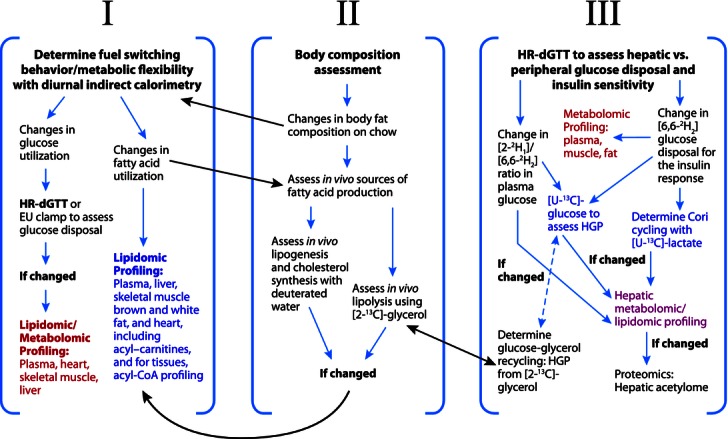
Framework for integrating fluxomic, metabolomic and lipidomic profiling. Our approach is to use fluxomics as a primary tool for metabolic phenotyping, and to layer additional omic information, such as metabolomics, lipidomics, and proteomics (acetylome determination), in a hypothesis-driven manner, and vice versa, to use fluxomics to elucidate the importance of other omic findings. (I) Discovery framework resulting from observing changes in fuel utilization with indirect calorimetry. (II) Discovery framework resulting from observing changes in body composition. (III) Discovery framework resulting from observing changes in flux measured via the hepatic recycling glucose (deuterated) tolerance test (HR-dGTT). The HR-dGTT yields information about peripheral and hepatic glucose disposal that can localize tissue specific metabolic/lipidomic screening. Changes in hepatic versus peripheral glucose disposal are assessed from the time course of percent differences in plasma [2-^2^H_1_]-glucose vs. [6,6-^2^H_2_]-glucose enrichments (1-ratio([2-^2^H_1_]/ [6,6-^2^H_2_])-glucose).^6^ The correlation with the hepatic global acetylome can be assessed in a hypothesis-driven framework, along with other fluxomic methodologies for assessing lipolysis (adipose), hepatic-adipose/glucose-glycerol recycling (HGP from [2-^13^C]-glycerol), lipogenesis, and Cori cycling (HGP from [U-^13^C]-lactate). The stable isotope tests shown are closed loop tests that are performed at the basal glucose and insulin levels (glycerol production and HGP), or dynamic tests incorporating the HR-dGTT and insulin responses. Closed loop tests do not require experimental groups to have identical, fixed values in glucose and insulin that are needed for open loop tests like the euglycemic hyperinsulinemic (EU) clamp.^7^ Tissue assessments in this framework assume an animal model. HGP, hepatic glucose production; D_2_O, deuterated water. Image courtesy of Irwin J. Kurland.

As an alternative to shotgun accumulation of large omic data sets, phenotypic data gathering can be done in a step-wise progression for hypothesis driven research. Irwin Kurland (Albert Einstein College of Medicine) presented a tiered framework in which commonly used measures of metabolism (e.g., phenotyping tests such as calorimetry and body composition analysis) and a novel deuterated glucose tolerance test (termed the hepatic recycling deuterated glucose tolerance test, or HR-dGTT) that assesses peripheral versus hepatic glucose disposal,^6^,^7^ are performed first to help determine which specific omics experiments to do next in animal models (Fig. 1). The results of each of these tests can inform subsequent experiments to generate a hypothesis-driven, multi-omic investigative framework.

If the measurements of fuel utilization by indirect calorimetry (Fig.1, panel I) indicate a change in carbohydrate or fat utilization, for example, plasma and muscle metabolomic and lipidomic profiling may be indicated. Or, if measurements of body composition reveals changes in body fat (Fig. 1, panel II), one could follow up by measuring lipogenesis using deuterated water^8^ or lipolysis using [2-^13^C]-glycerol.^8^–^10^ Changes in lipogenesis and/or lipolysis then provide enough evidence to follow up with lipidomic analyses, such as acyl carnitine or acyl CoA profiling,^8^ to monitor which lipids are being produced and/or broken down. The hepatic recycling glucose (deuterated) tolerance test (HR-dGTT, Fig. 1, panel III) assesses peripheral glucose disposal, as well as the recycling of glucose through the liver (a function of hepatic glucose uptake), based on plasma measurements that assess the decay in relative enrichment of administered [2-^2^H_1_]-glucose, versus [6,6-^2^H_2_]-glucose. Notably, while both [2-^2^H_1_]-glucose and [6,6-^2^H_2_]-glucose are taken up by the liver (via a process catalyzed by glucokinase); only [6,6-^2^H_2_]-glucose exits the liver unchanged after traveling through the glucose/glucose-6-P futile cycle (via a process catalyzed by glucose-6-phosphatase). However, there is substantial loss of [2-^2^H_1_]-glucose before exiting by exchange of the deuterium at the 2-position with water protons during the rapid equilibration of glucose-6-P with fructose-6-P, which does not affect hydrogens at carbon 6.^6^ Peripheral glucose disposal is estimated from the [6,6-^2^H_2_]-glucose area under the curve (AUC) during the HR-dGTT, and insulin AUC can also be obtained for an estimate of whole body insulin resistance. If changes in hepatic versus peripheral glucose disposal are observed by the HR-dGTT, additional stable isotope tests can be performed to monitor hepatic glucose production (HGP), lipolysis and glucose/glycerol recycling (by assessing glycerol production and HGP from glycerol), and glucose/lactate (Cori) re-cycling (by assessing lactate production and HGP from lactate) (Fig. 1). The results of these tests can coordinate tissue-specific metabolomic and lipidomic profiling efforts. Decisions can then be made to perform related global omic profiling, such as tissue-specific acetylome determination, thus leading to integrated omic information that may underlie diabetes development.

This sequential phenotyping paradigm has been applied to several mouse models,^6^–^17^ including a model of increased insulin sensitivity, the Pten^+/−^ mouse^6^ and a fatty acid amide hydrolase (FAAH)-knockout mouse, a novel model of the pre-diabetic state,^8^ which has reduced hydrolysis of endocannabinoids such as anadamide, and a type 2 diabetic mouse model, the MKR mouse.^10^ Pten normally inhibits insulin signaling by deactivating the product of insulin-stimulated phosphatidylinositide 3-kinases. Because insulin signaling stimulates hepatic glucose uptake, one might expect that the Pten^+/−^ mouse would show increased hepatic glucose uptake. However, the HR-dGTT revealed dramatically decreased hepatic glucose uptake in the Pten^+/−^ mouse, which correlated with decreased basal glucokinase expression, whereas HGP was the same as in the wild-type mouse. To explain these counterintuitive results, Kurland and collaborators hypothesized that, to ensure that enough glucose is supplied to the brain, hepatic glucose uptake is dramatically suppressed in the fasted state, leaving hepatic gluconeogenesis unaffected, so that hepatic glucose production occurs as normal. Glucokinase expression in the fasted to re-fed transition was markedly induced in Pten^+/−^ mouse livers,^6^ indicating increased insulin sensitivity, suggesting basal HGP regulation is under the control of factors besides insulin signaling, such as neural control.

The second model Kurland presented was the FAAH^−/−^ mouse.^8^ FAAH^−/−^ mice mimic several metabolic aspects of pre-diabetes, including obesity impaired fuel utilization, hyperinsulinemia, and insulin resistance in liver, skeletal muscle, and adipose tissue. The HR-dGTT indicated that the FAAH^−/−^mice had higher fasting insulin levels and higher blood glucose and insulin levels during the GTT even though glucose uptake in the periphery was the same as wild-type mice.^8^ The hyperglycemia was due, in part, to a non-suppressibility of HGP, indicated by the difference between the total plasma glucose (labeled and unlabeled) level during the HR-dGTT (higher for FAAH^−/−^) and the [6,6-^2^H_2_]-glucose level (unchanged versus wild type) during the HR-dGTT, as well as non-suppressibility of HGP (demonstrated with U^13^C glucose) during fasting. In accord with the stepwise phenotyping procedure described in Figure 1 (panels II and III), this led to examining the breakdown of lipids in adipose tissue (lipolysis), and HGP from glycerol as lipolysis of adipose triglycerides creates glycerol and fatty acids. Notably, administering [2-^13^C]-glycerol to mice and monitoring its dilution can provide information on how actively adipose tissue is breaking down lipids, and [2-^13^C]-glycerol can be followed to the production of ^13^C-glucose to assess HGP from the hepatic triose-P pool (Fig. 1). In FAAH^−/−^ mice, nonsuppressed and increased basal glycerol production and a corresponding increase in the use of glycerol for glucose production in the liver were observed. Metabolite profiling was then indicated (Fig. 1) and showed decreased triose-P metabolites in the fasted state of FAAH^−/−^ mice that support the re-direction of triose-P intermediates to the increased HGP from glycerol seen. FAAH^−/−^ mice also showed changes in TCA cycle metabolites that affect the malate–aspartate shuttle, one of the main conduits for transferring energy from glycolysis into the mitochondria. In particular, fasted citrate levels were decreased and fed citrate levels increased, indicating perturbations in acetyl CoA levels that were subsequently confirmed by direct measurements of acetyl carnitine and acetyl CoA measurements. This lead to the assessment of the fasted/fed hepatic acetylome following the framework shown in Figure 1.

Acetyl CoA sits on the crossroad of glucose, fatty acid, amino acid, and cholesterol metabolism, and so acetyl CoA has been proposed to be part of metabolic sensor and feedback mechanisms that regulate fuel utilization in the fasted and re-fed states (reviewed in Yang *et al*.^15^). The acetylome consists of proteins whose activities are regulated by acetylation, and this process relies on acetyl CoA as an acetyl donor. Changes in acetylation for mitochondrial malate dehydrogenase (MDH2), which was hypoacetylated in fasted FAAH^−/−^ livers, and hyperacetylated in fed FAAH^−/−^ livers, supports the metabolite profiling, indicating an impairment in the malate/aspartate shuttle. While dihydroxyacetone-P (DHAP) and glycerol-3-P levels were decreased in the fasted state of the FAAH^−/−^ mice, they were preserved in the fed state, consistent with a compensating contribution from a decrease in fed aldolase B acetylation in FAAH^−/−^ mice. These studies show how, by beginning with simple whole body measurements, such as calorimetry and measurements of body composition, one can eventually work towards understanding mechanisms at the molecular level.

## Tools for evaluating omics data

The complement to gathering omic data by applying the hypothesis-driven framework of Kurland and his collaborators (Fig. 1) is to gather and use omics data in an untargeted approach to try to generate novel hypotheses and to identify new targets that inform subsequent experiments. A challenge is that untargeted omics data collections can be difficult to analyze due to the sheer size of the datasets.

Charles Burant (University of Michigan Medical School) discussed two programs developed to visualize and analyze several types of omics data. The hope is that, by using these tools, researchers can generate hypotheses about the metabolic networks that respond to particular types of intervention, which can then be tested for their therapeutic value.

The first tool, Metscape 2,^18^ is a plugin for the program Cytoscape, a common platform for visualizing complex networks. Currently, Metscape 2 can incorporate gene expression and metabolomics data across different time points or different experimental conditions. Based on input data, Metscape 2 creates interaction maps that allow researchers to visualize the changes in gene expression and in metabolite levels in an attempt to link these changes to disease states. The second tool that Burant discussed was CoolMap (developed by colleagues G. Su and M. Fan), which enables researchers to visualize large, two-dimensional data and to interpret correlations between datasets. CoolMap can manage datasets of 8000 × 8000 data points and shows the Pearson's correlation coefficient in a heat map-like format.

**Figure 2:**
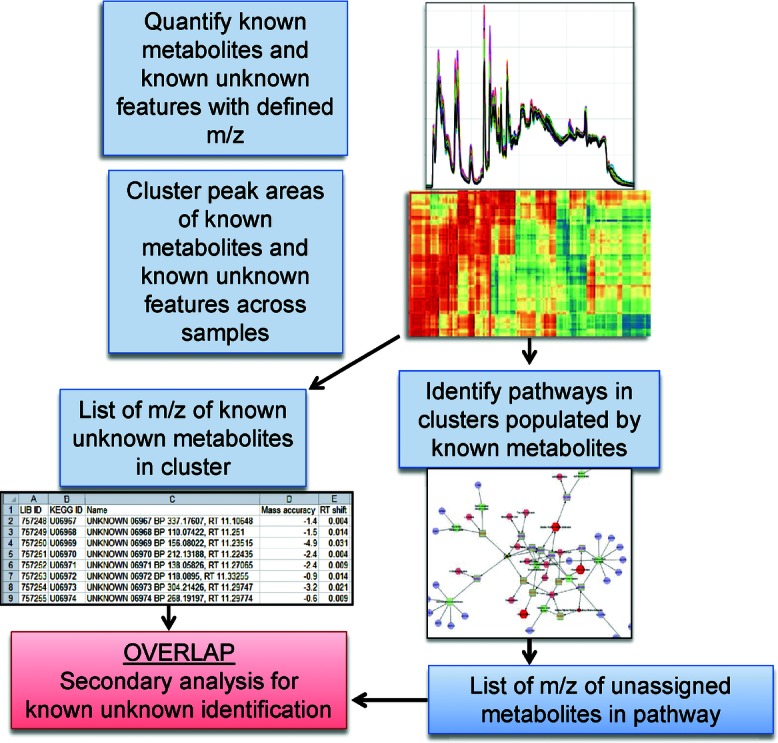
Schematic of Coolmap workflow to identify metabolic pathways. Automatic clustering of metabolite levels can identify metabolic pathways generated by known compounds contained in the clusters. ‘Known Unknown’ features with masses matching other metabolites in the identified pathway can aid in the identification of unknown metabolites. Image courtesy of Charles Burant.

As an example, Burant showed two CoolMap plots of various clinical parameters before and after weight loss. By visualizing the changes in these parameters, researchers can focus on the relationships that differ between the two states and can generate hypotheses that can be tested in further experiments. The usefulness of CoolMap will manifest in its ability to identify *known unknowns*, which are, according to Burant, unidentified, reproducible features in mass spectrometry data generated from untargeted high-throughput metabolomic studies (Fig. 2). To demonstrate this point, Burant used a CoolMap to show the correlation between various metabolites (fatty acids, amino acids, acetyl CoA, etc.) from a group of 25 people after the subjects had lost an average of 22.5% of their body weight. CoolMap can cluster the metabolites to reveal groups of metabolites that are highly related. By exporting a group of highly-related metabolites into Metscape 2, Burant showed that these metabolites were all part of a common pathway. Once a particular pathway is suspected of being important, researchers can hypothesize what other metabolites they should be able to see in their data and can go back to their original mass spectrometry data and identify some of their known unknowns.

While Burant and colleagues have already made Metscape 2 available to researchers (with CoolMap soon to be released), they are also constantly improving the platforms. Future versions of Metscape 2 should be able to integrate proteomic, phosphoproteomic, and acetylomic data to understand the relationship between genes, proteins, and metabolites in various states. Burant and co-workers are working to integrate CoolMap with Metscape 2 and with other omics programs to provide a suite of tools that integrate various types of directed (targeted) metabolomics, in which specific metabolites of interest are identified and quantified against stable isotope standards, as well as undirected (untargeted) metabolomics, in which researchers are not looking for specific metabolites but are instead performing an unbiased survey of which metabolites are sensitive to changes in various conditions.

## Branched chain amino acids

One of the goals of omics techniques, as described by Christopher Newgard (Duke University Medical Center), is to create metabolic signatures of human diseases that can be used as prognostic factors, to monitor disease progression, guide therapeutic interventions, and for hypothesis generation that can be tested in animal models. Newgard's talk included a comparison between targeted metabolic profiling of obese individuals with pre-diabetes and insulin resistance (a body mass index (BMI) of ∼36) to that of lean individuals (a BMI of ∼22).^19^ While previous studies have also looked at metabolic differences between obese and lean individuals, they have primarily focused on one or a small number of metabolites based on the particular hypothesis of each study. In contrast, Newgard's study gathered omic data to generate hypotheses using targeted metabolomics to measure over one hundred analytes.

After grouping the analytes of interest by principal component analysis (PCA), Newgard focused on one group—containing branch-chain amino acids (BCAAs-valine, leucine and isoleucine), glutamate and glutamine, 3- and 5-carbon acyl carnitines (C3-AC, C5-AC), and aromatic amino acids phenylalanine and tyrosine—that together explain most of the variance in the data. Most of these compounds are linked not just by PCA analysis but metabolically via BCAA metabolism. For example, isoleucine and leucine produce C5-AC in mitochondria from 2-methylbutyryl CoA and isovaleryl CoA, respectively. Glutamate is linked to the BCAAs via transamination in the cytoplasm. BCAAs go through a similar set of reactions during catabolism, which generate glutamate during a transamination first step in the cytoplasm; and C3-AC is generated from proprionyl CoA produced from valine and isoleucine metabolism. The aromatic amino acids phenylalanine and tyrosine may compete with the BCAAs for the same transporters to enter cells.

Other studies have shown an association between BCAA levels and insulin resistance; however, the advantage of Newgard's study is that because it was done by using an unbiased metabolomic analysis, the researchers were able to show that the whole pathway related to BCAA metabolism is elevated, and that the BCAA profile was the one most strongly associated with insulin resistance: interestingly, more so than the lipid-related signatures.

Newgard demonstrated that the BCAA/insulin resistance signature replicates in other cohorts with insulin resistance, for example, the Studies of a Targeted Risk Reduction Intervention through Defined Exercise (STRRIDE) trial^20^ and an Asian/Indian cohort.^21^ Also, the BCAA cluster of metabolite associations in interventions to treat diabetes, such as gastric bypass surgery and weight loss, predicts who will have an improvement in insulin sensitivity more effectively than the amount of weight actually lost.^22^ The decrease in BCAA levels corresponds to the efficacy of the interventions, and gastric bypass was associated with a greater decrease in BCAA than was matched weight-loss intervention.^23^ Importantly, BCAAs were shown to play a causative role in insulin resistance. Rats fed a high-fat diet supplemented with BCAAs spontaneously ate less food and weighed mildly less than rats fed a normal high-fat diet, but rats on both diets were equally insulin-resistant.^19^

**Figure 3:**
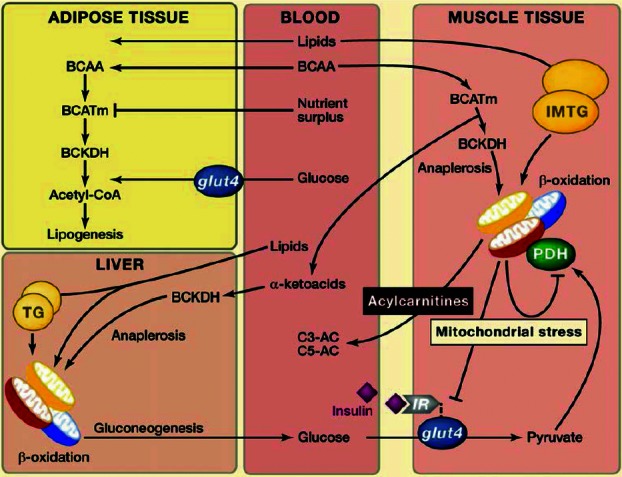
Schematic of a working model of potential crosstalk between lipids and branched chain amino acids (BCAA) in the development of obesity-related insulin resistance. *Anaplerosis* refers to repletion or filling up of TCA cycle intermediates via entry points other than acetyl CoA. TG, triglyceride; IMTG, intramyocellular triglyceride; IR, insulin receptor; BCATm, mitochondrial branched-chain aminotransferase; BCKDH, branched chain keto acid dehydrogenase; PDH, pyruvate dehydrogenase. Image courtesy of Christopher Newgard.

Newgard proposed a mechanism for the role of BCAAs in insulin resistance (Fig. 3) that centers on the role of inter-organ flux of BCAAs. Gastric bypass patients can have low expression of BCAA-metabolizing enzymes in adipose tissue, which increases after gastric bypass surgery and may explain the decrease in plasma BCAAs seen after gastric bypass surgery. In the setting of nutrient and caloric excess, which often occurs in a Western diet, the normal catabolism of BCAAs in adipose tissue is overwhelmed, and BCAAs exit into the bloodstream. These BCAAs find their way to muscle where they generate CoA species, such as succinyl CoA and proprionyl CoA, which enter the TCA cycle and impair the ability of mitochondria to completely oxidize fat. In the presence of these excess nutrients, the fuel-switching ability of the cell is impaired and glucose becomes almost superfluous as a fuel source, which could lead to the high blood glucose levels observed in pre-diabetes and a disturbance in metabolic fuel selection in diabetes even in the absence of impaired insulin signaling. The source of the BCAAs may also be related to the microbiome.

Barbara Kahn (Beth Israel Deaconess Medical Center and Harvard Medical School) followed up on the investigation of impaired BCAA metabolism in adipose tissue using a branched-chain aminotransferase (BCAT)-knockout mouse as a model. Knocking out BCAT impairs the ability to metabolize BCAAs and results in high serum BCAA levels. This state mimics characteristics of obesity, in which enzymes involved in BCAA metabolism are often downregulated, leading to high levels of circulating BCAAs. Replacing the adipose tissue in the BCAT-knockout mice with normal adipose tissue decreased the circulating levels of BCAAs, demonstrating that adipose tissue does indeed play a major role in regulating the levels of BCAAs.^24^

## Teasing the link between diabetes and obesity with mouse models

In addition to the work on BCAAs, Newgard presented data on using mouse models in an attempt to understand the link between obesity and diabetes. Starting with two common laboratory strains of mice, C57BL/6 and BTBR, Newgard, in collaboration with Alan Attie (University of Wisconsin), introduced the *ob* gene into these mice to create two distinct strains of genetically-induced obese mice. While both strains are insulin resistant, only one progresses to diabetes. Breeding these strains together and performing genomic and metabolomic profiling revealed gene–transcript–metabolite and gene–metabolite–transcript networks. Specifically, glutamate/glutamine (Glx) was significantly correlated to argininosuccinate synthetase 1 (Ass1), arginase 1 (Arg1), phosphoenolpyruvate carboxykinase 1 (Pck1), isovaleryl coenzyme A dehydrogenase (Ivd), and alanine:glyoxylate aminotransferase (Agxt) mRNAs.^25^ This is consistent with network models showing that quantitative trait loci (QTL) regulate Glx, which then regulates gene expression, or, conversely, QTL regulate mRNA abundance of the four transcripts, which then regulate Glx. These studies have the potential to uncover metabolic networks involved in the pathogenesis of diabetes.^25^

## Using omics to profile mechanisms for cardiovascular disease

Sixty percent of diabetics die from cardiovascular disease (CVD), and there is a four-fold increase in morbidity and mortality from atherosclerosis in the T2DM versus non DM population.^26^ Both Newgard and Domenico Accili (Columbia University) provided insight into the link between CVD and diabetes.

Why do some people with coronary artery disease experience cardiovascular events while others do not? To begin to address this question, Newgard turned to targeted metabolomic profiling. Elevated levels of short-chain dicarboxylacylcarnitines, ketone-related metabolites, and short-chain acylcarnitines were predictive of a composite endpoint of myocardial infarction (MI), repeat revascularization, or death at any point after coronary artery bypass grafting (CABG).^27^ A previous study had also shown that short-chain dicarboxylacylcarnitines were independently associated with a greater risk of death and incidence of MI for those undergoing cardiac catheterization.^28^

Dicarboxyl acyl carnitines seem to be predictive of subsequent CV events, and therefore Newgard and colleagues are now undertaking a two-pronged approach to further characterize the association of these metabolites with cardiovascular events. Using a human genetic approach, Newgard is performing metabolomic and genomic profiling of patients in Duke University's CATHGEN biorepository, which contains DNA and serum samples from people undergoing cardiac catheterization. To date, Newgard has profiled approximately 3500 individuals, 70% of whom have coronary artery disease and 30% of whom have diabetes. The genomic and metabolomic profiles of these patients have implicated genes involved in endoplasmic reticulum stress and in the unfolded protein response pathway. These genes are believed to play a role in modulating the concentrations of the small chain acyl carnitines.

## Coordinate regulation of cholesterol, bile acid, and lipid homeostasis via FoxO–FXR interactions

The crucible of interaction between diabetes and lipoprotein metabolism may be the liver, and the Accili laboratory has dissected biochemical pathways in liver that are regulated by nutrient and insulin signaling, dependent on the action of FoxO transcription factors. FoxO was previously thought of as a modulator of hepatic glucose production solely, and Accili presented new evidence linking dysregulation of FoxO action, which can stem from hepatic insulin resistance, to dysregulation of bile acid synthesis, leading to dysregulation of cholesterol synthesis and absorption and triglyceride synthesis, all of which can affect lipoproteins associated with an increased risk for cardiovascular disease. Bile acids are synthesized from cholesterol and have feedback effects that increase lipid and cholesterol absorption, lower plasma glucose, and decrease TG synthesis and levels. Bile acids act through their own subclass of orphan nuclear receptors FXRs, and the G protein–coupled bile acid receptor, TGR5. Bile acids are synthesized from cholesterol through both classical and alternative pathways. In the alternative pathway, the side chain oxidation of cholesterol precedes the steroid ring modifications, first yielding 24-, 25-, and 27-hydroxycholesterol metabolites, opposite to the process in the classical pathway. The alternative and classical pathway bile acids share the primary bile acid chenodeoxycholic acid, with 12α hydroxylation of chenodeoxycholic acid via CYP8B1 to cholic acid. Modifications of bile acids can affect their properties and their ability to activate these receptors. In mice lacking liver FoxO1 (L-FoxO1), 12-hydroxylated bile acids are reduced, while non-12-hydroxylated bile acids are increased. This is due to a sharp reduction in the expression of *Cyp8b1*, the gene encoding the 12-hydroxylase, in L-FoxO1 mice. The increase in hydrophilic (non-12-hydroxylated) over hydrophobic (12-hydroxylated) bile acids was shown to downregulate FXR, resulting in an increase in TG synthesis. The increase in hydrophilicity of the bile acid pool also contributed to an increase in cholesterol synthesis in L-FoxO1 mice, presumably a response to its contribution to low cholesterol absorption. This metabolomics analysis led to pursuing a testable hypothesis, would administration of a FXR agonist reverse the hypertriglyceridemia of L-FoxO1 mice?

L-FoxO1 and double mutant L-FoxO1:LDLR^−/−^ mice were used to test the role of FXR. Both mouse models were shown to have increased liver weight and TG content, and hypertriglyceridemia. When FXR ligand (GW4064 or cholic acid) is given to L-FoxO1 and to L-FoxO1:LDLR^−/−^ mice on a cholesterol-rich western diet for 8 to 10 weeks, FXR activation with cholic acid or GW4064 prevents liver TG accumulation in both mice strains, providing mechanistic evidence of the involvement of FXR.^29^

**Figure 4:**
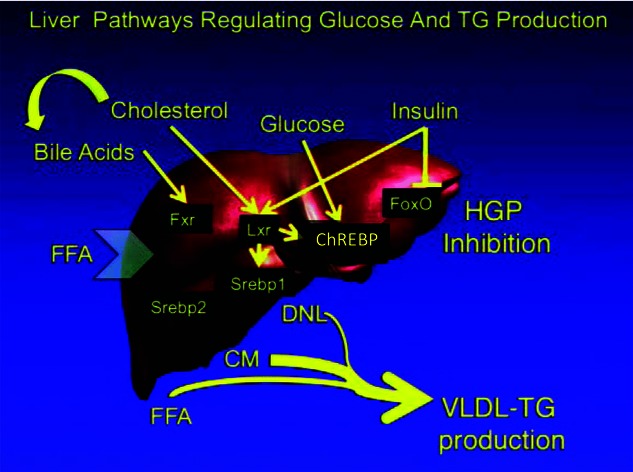
Outline of a working hypothesis for liver signaling pathways that affect diabetic dyslipidemia and hyperglycemia, linking bile acid, cholesterol, glucose, and insulin signaling to glucose, cholesterol, fatty acid, and triglyceride synthesis. FFA, free fatty acids; Srebp, Sterol regulatory element-binding protein; ChREBP, Carbohydrate-responsive element-binding protein; Lxr, Liver X receptor; Fxr, farnesoid X receptor**;** FoxO, Forkhead box O transcription factor; HGP, hepatic glucose production; DNL, *de novo* lipogenesis; TG, triglyceride; VLDL, very low density lipoprotein; CM, chylomicron. Image courtesy of Domenico Accili.

In summary, insulin, via FoxO, regulates the balance between 12-hydroxylated and non-12-hydroxylated bile acids. When non-12-hydroxylated bile acids are predominant in mice there is decreased FXR activation increased cholesterol, TG, and FFA synthesis and increased SREBP2 activation (Fig. 4). Based on these findings, Accili speculated that dyslipidemia in diabetes could be treated by targeting components of the bile acid synthetic pathway or by providing missing bile acids.

## Using omics to unravel the link between diabetes and the central nervous system

Both Accili and Gabriele Ronnett (Johns Hopkins University School of Medicine) discussed how metabolism in the brain affects food intake, energy utilization, and insulin sensitivity. The hormones insulin and leptin activate signaling pathways in the brain that decrease food intake and increase energy expenditure. However, under conditions of insulin resistance, these pathways are dysregulated. Studying how the brain regulates appetite and energy utilization identified candidate drug-susceptible targets that may be able to modify these processes during the course of diabetes.

## The role of FoxO1 in regulating appetite control in the brain

**Figure 5:**
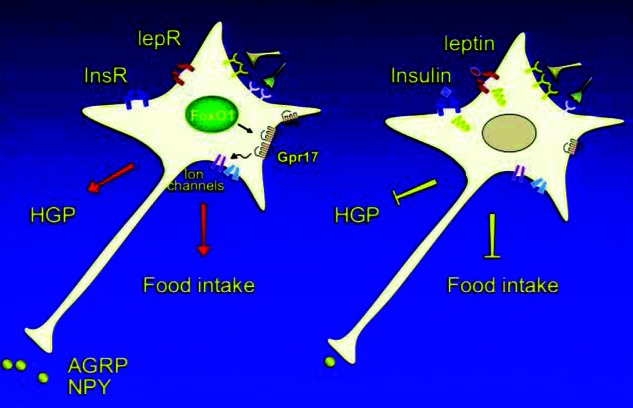
Model for regulation of food intake and hepatic glucose activity by FoxO1 and Gpr17. The G protein–coupled receptor Gpr17 is a FoxO1 target whose expression is regulated by nutritional status, and may play a role in mediating food intake. InsR, insulin receptor; lepR, leptin receptor; HGP, hepatic glucose production; NPY/AGRP, neuropeptide Y/Agouti-related peptide. Image courtesy of Domenico Accili.

In the brain there are two competing populations of neurons, those that make proopiomelanocortin (POMC) and those that make neuropeptide Y/Agouti-related peptide (NPY/AgRP), that compete for regulation of energy expenditure, food intake, and satiety. By activation of the catabolic POMC neurons, insulin and leptin decrease food intake and increase energy expenditure and physical activity. Such catabolic POMC neuron activity occurs concurrently with inhibition of the anabolic AgRP neurons, which, when activated, function to increase food intake and decrease energy expenditure and physical activity. Attempts to identify drugs that can modulate POMC and AgRP neurons have been fraught with difficulty. Genetic knockouts examining the role of these two subpopulations have been generally uninformative; for example, knockout of the insulin or leptin receptor in POMC or AgRP neurons has no apparent effect on food intake. FoxO1 is a shared mediator of both pathways and its inhibition is required to induce satiety. Fasting promotes FoxO1 nuclear localization in AgRP neurons, and whereas FoxO1 is excluded from the nucleus in the fed state. Accordingly, FoxO1 ablation in AgRP neurons of mice results in reduced food intake, leanness, improved glucose homeostasis, and increased sensitivity to insulin and leptin (Fig. 5). Peripherally, there is browning of white adipocytes in AgRP FoxO1 KO mice and evidence of increased mitochondrial size/mass (EM), along with increased expression of the mitochondrial uncoupling factor UCP1 in AgRP FoxO1 KO adipocytes. Importantly, knocking out FoxO1 from AgRP neurons increases the rate of glucose disposal and decreases HGP.

Integrated omic studies have been performed to identify the FoxO1 target in AgRP neurons. Signaling activation studies show pSTAT3 (a surrogate marker of leptin activation) is decreased and pAkt is increased in AgRP FoxO1 KO neurons. Immunohistochemistry showed pS6 signaling is increased in AgRP FoxO1 KO neurons projecting from the arcuate nucleus, signaling a state of abundant nutrients.^30^

Transcriptomic and electrical excitability studies indicate that AgRP FoxO1 KO neurons are less excited/more inhibited. Patch clamping shows FoxO1 KO AgRP neurons are constitutively inhibited. Expression profiling of flow-sorted FoxO1-deficient AgRP neurons identified an increase in GABA receptor (inhibitory) expression and a decrease in glutamate receptor (excitatory) expression, as well as the G protein–coupled receptor Gpr17 as a FoxO1 target whose expression is regulated by nutritional status (Fig. 5). Intracerebroventricular injection of Gpr17 agonists induces food intake, whereas the Gpr17 antagonist cangrelor curtails it. These effects are absent in AgRP-FoxO1 knockouts, suggesting that pharmacological modulation of this pathway, perhaps with brain-permeable Gpr17 agents, has therapeutic potential to treat obesity.^30^

## The role of fatty acid metabolism in the regulation of energy balance

**Figure 6:**
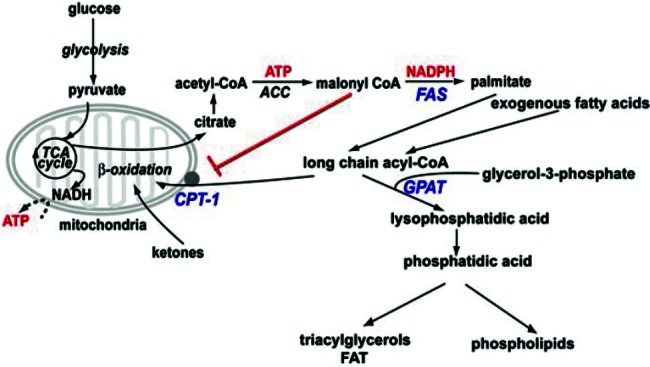
The role of fatty acid synthase (FAS), glycerol-3-phosphate acyltransferase (GPAT), and carnitine palmitoyl transferase 1 (CPT-1) in brain fatty acid metabolism. C75, an inhibitor of FAS and activator of CPT-1, FSG67, an inhibitor of GPAT, and C89b, an activator of CPT-1, all reduce food intake, increase energy expenditure, and enhance fatty acid oxidation to decrease adiposity and body weight. Image courtesy of Gabriele Ronnett.

The brain is a highly metabolic organ capable of fatty acid oxidation and storage, and the focus of Ronnett's group is the investigation of the hypothesis that pharmacological alteration of fatty acid flux can alter food intake. Ronnett's lab has focused on three metabolic enzyme candidates, key for the accumulation of long chain fatty acids, as targets for obesity intervention (Fig. 6). Fatty acid synthase (FAS) is a lipogenic enzyme that generates saturated long-chain fatty acids such as palmitate, which has 16 carbons. Carnitine palmitoyl-transferase-1 (CPT-1 isoforms) is requisite for the entry of long-chain fatty acids into mitochondria for oxidation. Glycerol-3-phosphate acyltransferases (GPATs) catalyze the first and rate-limiting step for fatty acids to phospholipid and triglyceride syntheses. In general, increased fatty acid oxidation is characteristic of the fasted state, and Ronnett hypothesized that either FAS or GPAT inhibition, or CPT-1 stimulation in the central nervous system, would decrease food intake and body weight. Ronnett tested this hypothesis using three small molecules: C75, an inhibitor of FAS and activator of CPT-1; FSG67, an inhibitor of GPAT; and C89b, an activator of CPT-1; and confirmed that these molecules reduce food intake, increase energy expenditure, and enhance fatty acid oxidation to decrease adiposity and body weight.^31^–^34^

Central administration of these compounds alters neuronal activity in select hypothalamic nuclei that control food intake and energy expenditure. In these hypothalamic nuclei, compound treatment leads to altered gene expression and production of neuropeptides germane to energy balance, consistent with homeostatic responses to CNS perception of physiologically positive energy balance.

Recent work has shown that obeseogenic diets high in saturated fatty acids, known to cause peripheral inflammation and exacerbate diabesity, also induce CNS inflammation, ER stress, and oxidative stress, and that this may contribute to the development of metabolic syndrome.

Both C75 and FSG67 induced weight loss in obese mice. Examining the genetic effects of these compounds revealed that the synthesis of enzymes involved in fatty acid storage was downregulated, whereas the synthesis of enzymes involved in fat disposition was upregulated. FSG67 is currently in preclinical safety tests. C89b, the CPT-1 stimulator, also decreased food intake and induced weight loss, consistent with the role of CPT-1 in promoting lipid oxidation. The effects seen with C89b in mice, however, were more dramatic and longer-lasting than those seen with C75 or with FSG67.

To investigate the mechanisms of these compounds’ effects, researchers are conducting metabolomic studies in neurons *in vitro*. So far, they have seen that C75 and FSG67 increase reactive oxygen species while reducing the secretion of inflammatory cytokines. Based on these data, Ronnett speculated that C75 and FSG67 are not just altering fatty acid metabolism in the neurons but may also have long-term effects on inflammation in the brain. To understand what other pathways are affected by alteration of fatty acid flux and to elucidate what metabolic changes are affecting the observed changes in inflammatory signals, Ronnett is undertaking a full metabolomic profile in primary hypothalamic and cortical neurons treated with palmitate, C75, and FSG67. Initial results indicate that primary hypothalamic neurons show different responses to these agents. Under normal nutrient conditions, hypothalamic neurons did not have a significant fatty acid profile response to C75; however, in the setting of nutrient (palmitate) excess, C75 did have a significant effect and caused a decrease di- and triglycerides in primary hypothalamic neurons. FAS inhibition did increase TCA metabolite levels, which suggests a pathway for modification of ATP levels other than manipulation of fatty acid oxidation, which may be part of an AMPK related mechanism of action for these agents.^35^

## Targeted versus global untargeted metabolomics profiling as a tool for metabolic phenotyping

An industry panel—Steven Fischer (Agilent Technologies), Suma Ramagiri (AB SCIEX), John Ryals (Metabolon), Mark Sanders (Thermo Fisher Scientific), John Shockcor (Waters Corporation), and Joe Shambaugh (Genedata)—was asked to discuss the benefits, drawbacks, and areas of future development for targeted versus global untargeted profiling as tools for metabolic phenotyping.

In general, mastering the tools of chromatographic separation methods takes precedence over metabolite identification. Normal phase and reversed phase chromatography have synergism for global small molecule separation and identification.^36^ Supercriticial fluid chromatography, for example, may provide a new modality for future global lipid analysis.^37^ Derivatization can aid targeted LC/MS/MS analysis, as used in amino acid^38^,^39^ and acyl carnitine analysis.^40^,^41^

**Figure 7:**
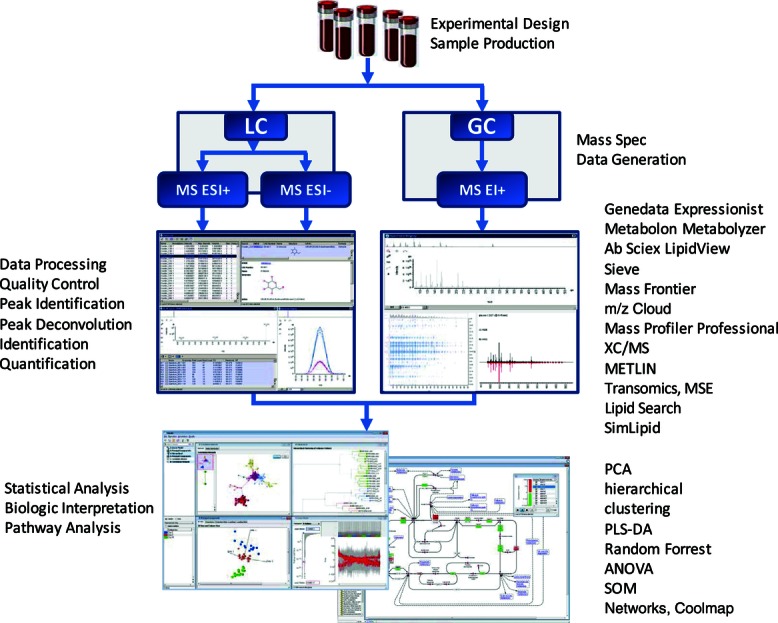
Overview of metabolomic data generation and data analysis. The flowchart used for metabolite extraction, data mining, and metabolite identification is detailed. This illustrates sample preparation, mass spectrometric analysis, peak extraction/identification and compound quantification, and statistical data analysis for biomarker identification and mapping of biomarkers to metabolic pathways. Samples, in general, can be divided into separate groups for gas chromatography/ mass spectrometry (GC/MS) and liquid chromatography/MS (LC/MS). LC/MS is further divided to examine both positively and negatively charged ions, first done with full scan for exact mass, and after with LC/MS/MS for identification with databases such as METLIN, SimLipid, LipidView, Lipid Search or Mass Frontier, or in the case of GC/MS, Fiehn and NIST libraries. *Data preprocessing* covers the software programs that process complex raw data to clean data. Data preprocessing programs (for example, Genedata Expressionist for Mass Spec, Transomics, XC/MS, Sieve, Metabolon Metabolyzer) are used to separate peaks from noise, and then database searching can be accomplished. Genedata Expressionist *for Mass Spec*, Transomics, and XC/MS are mass spectrometer platform–independent. A variety of techniques can be used for statistical analysis, including principal component analysis (PCA), partial least squares discriminant analysis (PLS-DA), analysis of variance (ANOVA), Random Forrest, self-organizing maps (SOM), and platform-independent software such as SIMCA-P, Transomics and Genedata and Expressionist *for Mass Spec* can be used for such analyses. An overview of such statistical methods can be found in Madsen *et al*.^57^ Image courtesy of Joe Shambaugh.

A targeted quantitative approach, using GC/MS and LC/MS/MS, is the best first approach for any metabolomics/lipidomics problem. This should be followed with a global profiling paradigm, first aimed at getting the best possible exact MS data, in particular with retention time locked databases, subsequently re-run to obtain MS/MS to aid database searching. The principal challenges in global profiling are the creative use of algorithms for the separation of peaks from noise, optimal data mining paradigms and databases, and for biofluids determining the source for the metabolites identified^42^–^47^ (Fig. 7).

Metabolite biomarkers include those synthesized *in vivo* and those derived from exogenous sources, including the microbiome. The consensus was that humans are capable of synthesizing roughly 2500 compounds. As reviewed in Dunn *et al*.,^48^ 2,000–7,000 metabolic features can be detected in a serum or plasma sample. A single metabolite can be detected as different ion types: for example, as protonated and deprotonated ions, adduct ions, isotopomers, fragment ions, dimers, and trimers. Therefore, a large number of metabolic features identified correspond to a smaller number of actual metabolites.^49^ Humans may contain more molecules than they are able to directly synthesize, due to microbiome metabolism, drugs, or dietary supplements. Differences in the amount of compounds in human plasma found at different facilities stem, in part, from whether pooled human samples were used versus individual test subjects, as well as some differences due to the particular MS platform used. Pooled plasma samples have as many as 2000 compounds (Fischer, private communication), while individual subjects have at least 500–600 compounds.^50^

The dataset derived from untargeted mass spectrum analysis may be very noisy, with noise in unit mass and/or accurate mass instruments being ∼80% of the total data collected.^51^ Optimal peak identification/separation of sample peaks from chemical noise, and clustering of their GC/MS and LC/MS data before library search for metabolite identification, is facilitated by software packages such as Mass Profiler Professional, Thermo Scientific Sieve,^52^ Genedata Expressionist for Mass Spec,^53^ Transomics, and XC/MS^45^ (see Fig. 7).

The current data mining paradigm involves extracting data using a naive feature extractor and performing compound identification on the reconstructed spectra. Untargeted mass spectrum analysis is facilitated by assembly of a database composed of a large number of library standards. Each standard entry can have a number of features, such as a retention time index, MS spectra, and MS/MS fragmentation spectra, obtained at different collision energies. Retention time libraries can be machine- and column-specific, as different machines have different sensitivities, and some problems requiring nano-UPLC will necessarily have a different retention time library than standard UPLC. As mentioned, due to the redundancy of the ion spectra, each library entry may have ∼ 10 or more features, as each molecular standard can be associated with 5–10 ion features.^48^,^51^,^54^ The current Agilent-METLIN database and MS/MS library contains ∼ 45,000 compounds, with ∼ 9000 compounds having MS/MS spectra.^44^ METLIN data has been acquired using a collision cell shared by triple quadrapole and qTOF machines. MS/MS spectra are collected in both positive- and negative-ion mode and at 10, 20, and 40 eV collision energies. Those spectra that have at least one ion with ∼ 1000 counts of signal are retained for entry into the MS/MS library. The spectra are edited to only include ion signals coming from the standard, and the reported mass is corrected to its theoretical mass.^44^ GC/MS metabolite identifications are facilitated by well-defined MS conditions and libraries, as reviewed Kind and Fiehn,^55^ and METLIN, Mass Frontier, and m/z Cloud^a^ are establishing databases that together cover a wide variety of MS platforms.

The loose fit of MS^n^ spectra with the METLIN database suggests that MS and MS^n^ spectra generated on LTQ-Orbitrap machines are best identified by Mass Frontier.^55^ The larger the database, the better it works, and the m/z Cloud community-based effort aims to establish a comprehensive library of high quality spectral trees to improve the structural elucidation of unknowns by identifying compounds even when they are not present in the library, using spectral tree searches. For example, individual MS^n^ spectra can be searched against the m/z Cloud library to retrieve structural or substructural hits. The challenge is reassembly, which can be expert-motivated and have input from correlations with other metabolites to assemble the puzzle.^55^

Lipidomic database searches benefit from the LipidMaps initiative.^56^ which has resulted in dedicated commercially available *in silico* lipid databases such as LipidView (Ab Sciex), SimLipid (Biosoft), or Lipid Search (MKI), enabling one to uniquely identify over 20,000 lipid species using characteristic lipid fragments.^50^

The use of pathways as a means to interpret metabolomics data acquired using non-targeted data acquisition strategies opens up a different approach to data mining. By using pathways for biological interpretation, the researcher has defined the metabolites in the pathway(s) as a target compound list. The identified target list then can be used for statistical analysis^57^ (Fig. 7) rather than just analyzing features. This compound list can be used as the template for further mining the pathway(s) using targeted identification and data extraction.

Future developmental work could center on matching possible metabolites at successive nodes, integrating searching with pathway databases for both GC and LC. For GC, this would involve theoretical calculations of derivatization effects.^58^ As compounds are actually identified, a database can be created that records this information for future use in compound identification. Another possibility is to use genome-wide association studies (GWAS)^59^ data to see if there is an association to the molecule of interest. At times, associating a particular allele to specific metabolite biomarkers may suggest a known gene or a gene of a known class.

An unknown compound can be identified, tracked, and quantitated with relative or semi-quantification even though its true identity is not known. If such a molecule becomes an important biomarker, there are several approaches that can be used to either suggest an identity or get clues as to the identity. Biochemicals are typically not independent variables; they change in groups that are related biosynthetically or functionally, and statistical correlative methods can be of use to postulate relationships. Important biomarkers identified in this manner can have their mass accurately determined, atomic composition calculated, and identification made more complete by using MS^n^ analysis. Such approaches can give scientists better ideas about the identity of the metabolite, its molecular composition, and the pathways involved in its metabolism.

## Conclusion

Metabolomics, lipidomics and fluxomics technologies are still in their relative infancy for general biomarker discovery, and can be integrated with other omics (proteomic, transcriptomic and genomic) to reveal a more complete picture of diabesity disease mechanisms. Complementary approaches to multi-omic metabolic pathway analysis may involve a tiered hypothesis-driven framework, to determine whether another omic may be indicated. Additionally, emerging visualization tools for shotgun omic data evaluations allow the generation of hypotheses about the metabolic networks that respond to particular types of intervention. Fluxomics and both targeted and global untargeted metabolomics profiling can be used, in conjunction with mouse dietary and genetic models, and human clinical studies, to unravel the link(s) between diabetes and obesity, and to profile metabolic mechanisms in and between the CNS and periphery (liver, fat, muscle) that may affect plasma metabolic biomarkers. In general, metabolomics, lipidomics and fluxomics hold the promise not only for diagnostic evaluation in routine clinical use to predict disease progression and outcomes, but also, by nature of their pathophysiological relevance, for identification of target pathways that may relate to molecular mechanisms. Identified pathways then can be the focus of drug development for future use in therapeutics for personalized medicine.

## Footnotes

a http://www.mzcloud.org/

## Appendix

### Additional reading

Newgard C.B. 2012. Interplay between lipids and branched-chain amino acids in development of insulinresistance. *Cell Metab*. **15:** 606–614

Sana T.R., K. Waddell & S. M. Fischer. 2008. A sample extraction and chromatographic strategyfor increasing LC/MS detection coverage of the erythrocyte metabolome. *J. Chromatogr. B*. **871:**314–321

Sana T.R., J.C. Roark, X. Li, *et al*. 2008. Molecular Formula and METLIN Personal Metabolite DatabaseMatching Applied to the Identification of Compounds Generated by LC/TOF-MS. *J. Biomol. Tech*. **19:**258–266

Adamski J. & K. Suhre. 2013. Metabolomics platforms for genome wide association studies-linking thegenome to the metabolome. *Curr. Opin. Biotechnol*. **24:** 39–47
